# The Prevalence and Endemic Nature of Dengue Infections in Guangdong, South China: An Epidemiological, Serological, and Etiological Study from 2005–2011

**DOI:** 10.1371/journal.pone.0085596

**Published:** 2014-01-23

**Authors:** Ru-ning Guo, Jin-yan Lin, Lin-hui Li, Chang-wen Ke, Jian-feng He, Hao-jie Zhong, Hui-qiong Zhou, Zhi-qiang Peng, Fen Yang, Wen-jia Liang

**Affiliations:** 1 Public Health Emergency management office, Center for Disease Control and Prevention of Guangdong Province, Guangzhou, China; 2 Center for Disease Control and Prevention of Guangdong Province, Guangzhou, China; 3 Institute of Pathogenic Microorganisms, Center for Disease Control and Prevention of Guangdong Province, Guangzhou, China; 4 Institute of Infectious Disease Prevention and Control, Center for Disease Control and Prevention of Guangdong Province, Guangzhou, China; Pennsylvania State University College of Medicine, United States of America

## Abstract

**Objectives:**

Frequent outbreaks of dengue are considered to be associated with an increased risk for endemicity of the disease. The occurrence of a large number of indigenous dengue cases in consecutive years indicates the possibility of a changing dengue epidemic pattern in Guangdong, China.

**Methods:**

To have a clear understanding of the current dengue epidemic, a retrospective study of epidemiological profile, serological response, and virological features of dengue infections from 2005–2011 was conducted. Case data were collected from the National Notifiable Infectious Diseases Reporting Network. Serum samples were collected and prepared for serological verification and etiological confirmation. Incidence, temporal and spatial distribution, and the clinical manifestation of dengue infections were analyzed. Pearson's Chi-Square test was used to compare incidences between different age groups. A seroprevalence survey was implemented in local healthy inhabitants to obtain the overall positive rate for the specific immunoglobulin (Ig) G antibody against dengue virus (DENV).

**Results:**

The overall annual incidence rate was 1.87/100000. A significant difference was found in age-specific incidence (Pearson's Chi-Square value 498.008, P<0.001). Children under 5 years of age had the lowest incidence of 0.28/100000. The vast majority of cases presented with a mild manifestation typical to dengue fever. The overall seroprevalence of dengue IgG antibody in local populations was 2.43% (range 0.28%–5.42%). DENV-1 was the predominant serotype in circulation through the years, while all 4 serotypes were identified in indigenous patients from different outbreak localities since 2009.

**Conclusions:**

A gradual change in the epidemic pattern of dengue infection has been observed in recent years in Guangdong. With the endemic nature of dengue infections, the transition from a monotypic to a multitypic circulation of dengue virus in the last several years will have an important bearing on the prevention and control of dengue in the province and in the neighboring districts.

## Introduction

Dengue is a mosquito-borne infectious disease caused by 4 distinct, but closely related serotypes of the dengue virus (DENV-1, 2, 3, 4). Coinciding with the distribution pattern of its mosquito vectors, dengue has been reported as endemic in over 100 tropical and subtropical countries of the world [1], [2]. The World Health Organization currently estimates about 2.5 billion people at risk of dengue infection globally.

For an area that experiences dengue epidemics, there are often 2 patterns of infection and transmission: endemicity (one or multiple serotypes present) and non-endemicity (no virus sustained). An endemic area often has the following common features: young age groups at a greater risk of infection [3]–[5], co-existence of multiple serotypes of dengue virus in local areas [2], [3], [5], [6], a higher seroprevalence of DENV antibodies (as high as 80%) in local inhabitants [3], [7] compared to that in non-endemic regions [8], [9], and a continuous spectrum of dengue severity identified, with children often at a higher risk of developing a severe form [3]–[5], whereas travelers often experience typical or mild dengue fever [10], [11].

Guangdong province is located in South China, with a hot and humid sub-tropical weather. It has the highest incidence of dengue in mainland China [12]. Since the first laboratory-confirmed DENV-4 epidemic in Fo-shan of Guangdong in 1978 [12], [13], periodical infections and transmission of all 4 serotypes of dengue have been recorded in the past 30 years [12]. However, DENV-1 has become the most prevalent serotype in circulation since 1990 [12], causing epidemics and outbreaks in 1991 and from 1995–2010.

Although affected localities seemingly varied alternatively by year [14], [15], frequent outbreaks may influence the transmission dynamics and facilitate the endemic process [12], [16]. Epidemiological and limited phylogenetic analysis of virus isolates from 1979–2005 showed that dengue epidemics in Guangdong were closely associated with those in Southeast Asian countries, especially Philippines, Indonesia, and Thailand, indicating that dengue infections in Guangdong were still largely triggered by cases imported from overseas [14], [17], [18].

However, the circulation of DENV-1 over consecutive years in Guangdong reminds us of the possibility of a changing profile of dengue epidemic and endemicity in Guangdong as a large number of locally acquired dengue cases were consistently reported among the inhabitants [19]–[21]. The transition of a dengue epidemic pattern from non-endemic to hypo-endemic (one serotype present), or even hyper-endemic (multiple serotype present), might have been underway in Guangdong [21]. Evidenced-based epidemiological, serological, and virological studies are needed to illustrate this issue.

As a notifiable infectious disease in China, the prevention and control of dengue has been given high priority in Guangdong since 1978. Nevertheless, routine active surveillance was not conducted until 2003, and prior to 2003, dengue control relied almost solely on subsequent vector control and passive reporting and management of the patients. In 2005, virus monitoring in patients and serology surveillance targeted at healthy populations were initiated. On the basis of the surveillance results from the last 8 years in combination with earlier findings, our study aims to improve our understanding of the current epidemiological characteristics of dengue infections in Guangdong, South China, to evaluate the endemicity issue of dengue that has been posed by researchers since 2005.

## Materials and Methods

### Study area

Guangdong province is located in South China with typical hot and humid sub-tropical weather. It has an area of 179.8 thousand square kilometers and is divided into 4 regions: Pearl-River-Delta Area (PRDA, containing 10 prefectures), East Area (EA, containing 4 prefectures), West Area (WA, containing 3 prefectures), and Mountain Area (MA, containing 5 prefectures). Comprising a population of 104.3 million people, including millions of foreigners (2010 census data), and being located adjacent to Hong Kong and Macao Special Administrative Region, Guangdong has frequent economic and cultural communication with Southeast Asia and countries bordering the Western Pacific Ocean. Being a coastally developed district, Guangdong is also the transportation hub between mainland China and abroad. Therefore, Guangdong, with the most densely populated urban environment and a largely mobile population, has the natural and social conditions for mosquito vector breeding and reproduction. *Aedes albopictus*, as the primary vector, occupies the majority of the prefectures and counties in Guangdong, while the secondary vector—*Aedes aegypti*—only occupies one prefecture of Guangdong [12].

### Case data collection and specimen detection

Data from dengue cases from 2005–2011 were collected from the National Notifiable Infectious Diseases Reporting Network that was established in 2003. A suspected case was identified either by clinical doctors in medical institutions or by field epidemiologists during active case searching when outbreaks or epidemics occurred. Serum samples were collected and sent to the local prefectural Center for Disease Control and prevention for further serology verification and/or etiology confirmation. For acute-phase sera collected within 5 days of onset, TaKaRa One Step Prime Script TM reverse-transcriptase polymerase chain reaction (RT-PCR) kit (Perfect Real Time) was used for nucleic acid detection, and C6/36 or Baby Hamster Kidney (BHK21) cells (Shanghai Bioleaf Biotech Co., Ltd, China) were used for virus culture and isolation. RT-PCR was used for further subtype identification. The detection step was performed according to the manufacturer instructions. For sera collected beyond 5 days of onset, a dengue immunoglobulin (Ig) M/IgG enzyme-linked immunoassay kit (Zhong-shan Biological Engineering Co., Ltd., China) was used for antibody IgM/IgG testing. Convalescent sera (10–14 days) were also required for IgM/IgG verification.

A face-to-face case survey with a detailed structured questionnaire was administered to each suspected patient by well-trained field investigators to obtain the following information: age, sex, occupation, address, exact dates of onset and sampling, clinical presentation and duration, travel history, and mosquito bite exposure 2 weeks prior to symptom onset. When clusters of cases outnumbered 50, for practical purposes, the index case (clinically or epidemiologically), severe cases, and the first 50 patients of a cluster were administered the detailed questionnaire survey, and additional patients received a simpler form of the questionnaire that only obtained the primary demographic, clinical, and epidemiologic exposure information, according to the Provincial Dengue Surveillance and Prevention and Control Guideline.

A suspected, probable, or confirmed case of dengue was determined according to Dengue Diagnostic Criteria enacted by the China Health Criteria Committee in 2005, as outlined below. I. A suspected case is defined by the presence of clinical manifestations and epidemiologic exposure history (stay and mosquito bite history in the dengue-affected area within 2 weeks before the onset of illness). II. A probable case of dengue is defined as a suspected case meeting with the following 2 conditions: low white blood cell and platelet counts, and single serum positive for specific IgM or IgG antibodies. III. A confirmed case of dengue is defined as a probable case presenting with any of the following lab test results: a 4-fold increase in specific IgG antibody titer, positive result on a PCR test or a virus isolation and identification test. In addition, an outbreak is confirmed as the occurrence of 3 epidemiologically linked cases within 2 weeks in a limited area, according to the National Dengue Surveillance and Prevention and Control Guideline. To distinguish cases with different origins of infection, an imported case was defined as a patient who recently travelled to dengue epidemic/endemic countries 2 weeks prior to symptom onset. An indigenous case was defined as a patient whose source of infection was clearly in local areas within China, as opposed to imported cases.

### Seroprevalence study in healthy population

To assess the extent of dengue virus exposure in the population, a seroprevalence survey was conducted in healthy inhabitants from 2003 to 2011. Taking into consideration the infection history, and simultaneously accounting for other factors, such as geographical location, economic development, degree of urbanization and adherence to a surveillance program, 4 sentinel cities in Guangdong province were selected, including Guangzhou and Zhong-shan city in PRDA, Zhanjiang city in WA, and Shantou city in EA, which are all high-risk areas for dengue epidemic. With an expected seroprevalence of 5% (range, 1.1%–7.7%) in a non-endemic or hypo-endemic dengue area [9], [22], [23], an allowable error of 25%, and a significance level of 0.1, a sample size of 800 sera was predicted to be necessary by the simple random sampling method.

The seroprevalence survey was conducted twice a year, in April–May (pre-epidemic) and in December–January (after-epidemic). The epidemic season for dengue was considered to be July–October, during which time a strong association was observed between vector mosquito density and dengue incidence [14].

Two hundred sera samples from healthy local inhabitants (with no specific age requirements) were randomly collected during physical examination of the population in community clinics each sentinel year. A total of over 800 specimens were prepared and analyzed for specific IgG antibodies against DENV throughout recent years (except in 2003 and 2004, as a standardized surveillance was not performed until 2005). An IgG ELISA Kit (Zhong-shan Biological Engineering Co., Ltd., China) was used to test for the IgG antibody targeting serotypes 1, 2, 3, and 4. The sensitivity and specificity of the IgG ELISA Kit was determined to be over 95% [24]. The detection step was performed according to the manufacturer instructions.

### Statistical analysis

The database of dengue cases in 2005–2011 was set up using Epi data software and was used for descriptive epidemiological analysis. The annual incidence of dengue infections was obtained by using new cases of dengue identified that year as the numerator and mid-year population in the corresponding year as the denominator. Age-specific cumulative incidences were calculated by using the total new cases identified during 2005–2011 as the numerator, and the mean population during the period as the denominator. Pearson's Chi-Square test was used to compare incidences between different age groups. The positive rate for specific IgG antibodies against DENV was calculated.

### Ethical statements

The ethical review committees of the Center for Disease Control and Prevention of Guangdong province granted the ethical approval for this study. Under the premise of ensuring no violation of the Declaration of Helsinki for the protection of human subjects, oral informed consent was obtained from each suspected dengue patient whose serum sample was collected. For the suspected patients who were minors/children, informed consent was obtained from their guardians (parents, next of kin, or caretakers). An oral consent form was adopted, as the collection of sera samples is the routine and necessary clinical diagnostic test for dengue infections, as well as a normal public health response. Participants fully understood the significance of the blood collection procedure and its implied risks, as directly reflected and documented in the corresponding case investigation form. Written informed consent was obtained from each subject who participated in the seroprevalence study for the dengue IgG antibody. The whole process, along with the case investigation, did not reveal the participants' personal information; patients' privacy, confidentiality, rights, and interests were not violated.

## Results

### Age-specific incidences: no higher incidence found in younger individuals

**Figure 1:**
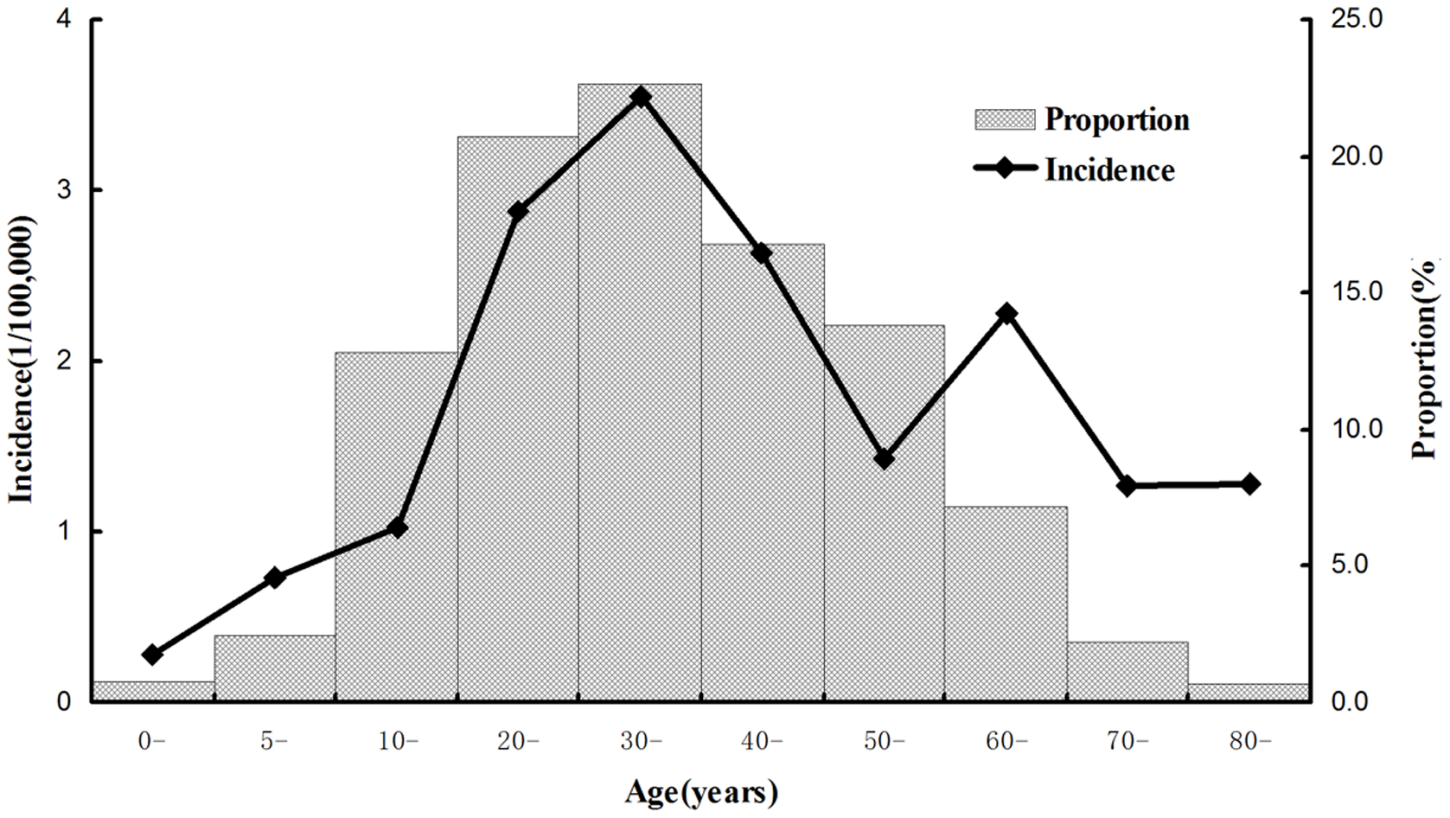
Age-specific incidence of dengue infections in Guangdong, China, 2005–2011. Incidences were indicated with the line chart. The proportion of age-specific cases to the total number was shown with the bar chart.

A total of 1779 dengue cases were reported between 2005 and 2011. Teenagers and adults aged 10–59 years accounted for 86.73% of the total patients (1543/1779). The overall incidence rate was 1.87/100,000. A significant difference was found between age-specific incidences (Pearson's Chi-Square value, 498.008; P<0.001). Adults 30–39 years of age had the highest incidence of 3.55/100,000, followed by those 20–29 years old (2.87/100,000), 40–49 years old (2.63/100,000), and 60–69 years old (2.28/100,000). Young children under 5 years of age had the lowest incidence of 0.28/100,000 (Figure 1).

### Temporal and spatial patterns: co-existence of imported cases and indigenous cases

**Figure 2:**
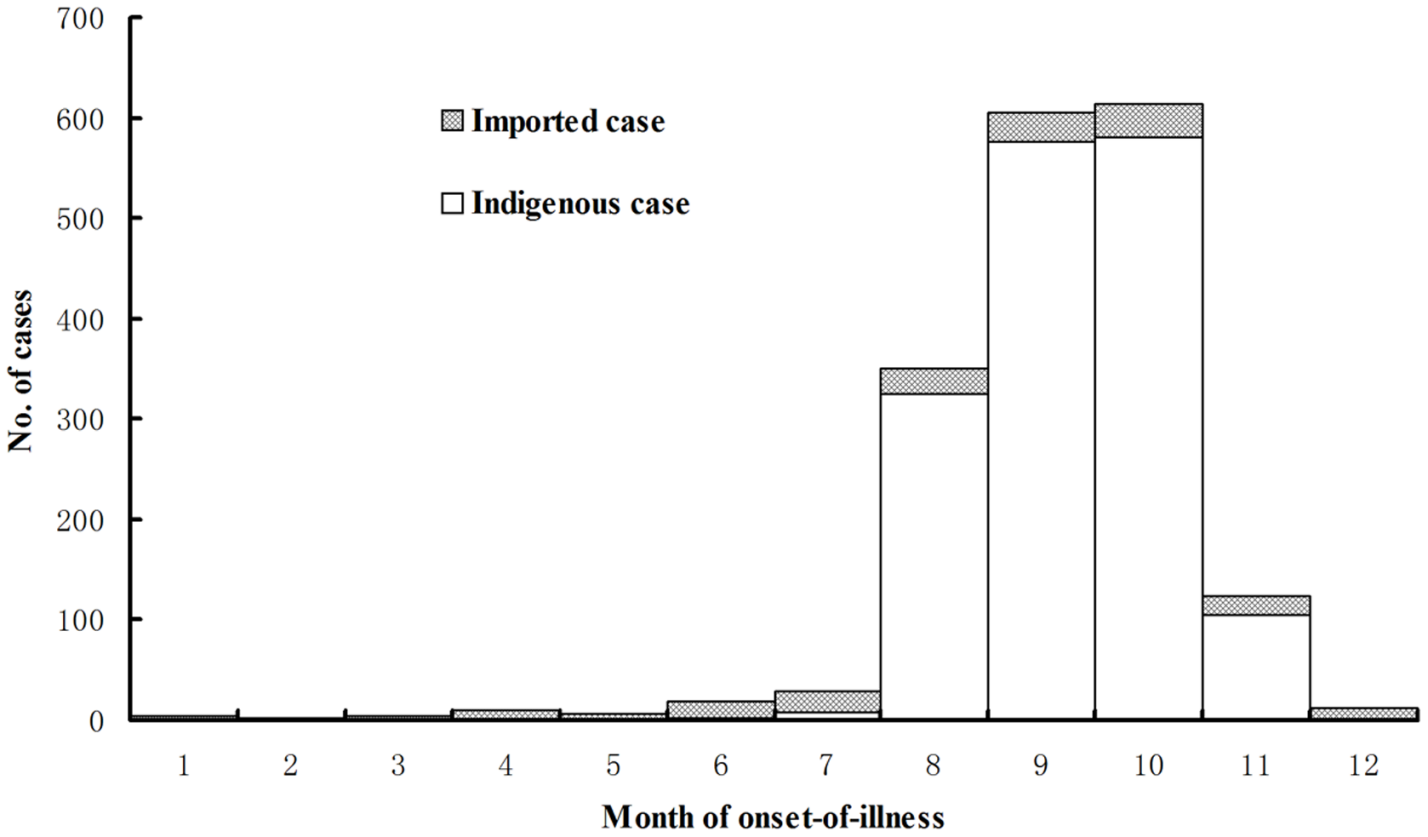
Onset month of dengue cases by infection origin in Guangdong, China, 2005–2011. Imported and indigenous dengue cases were shown with the stacked bar chart using different filling patterns.

**Figure 3:**
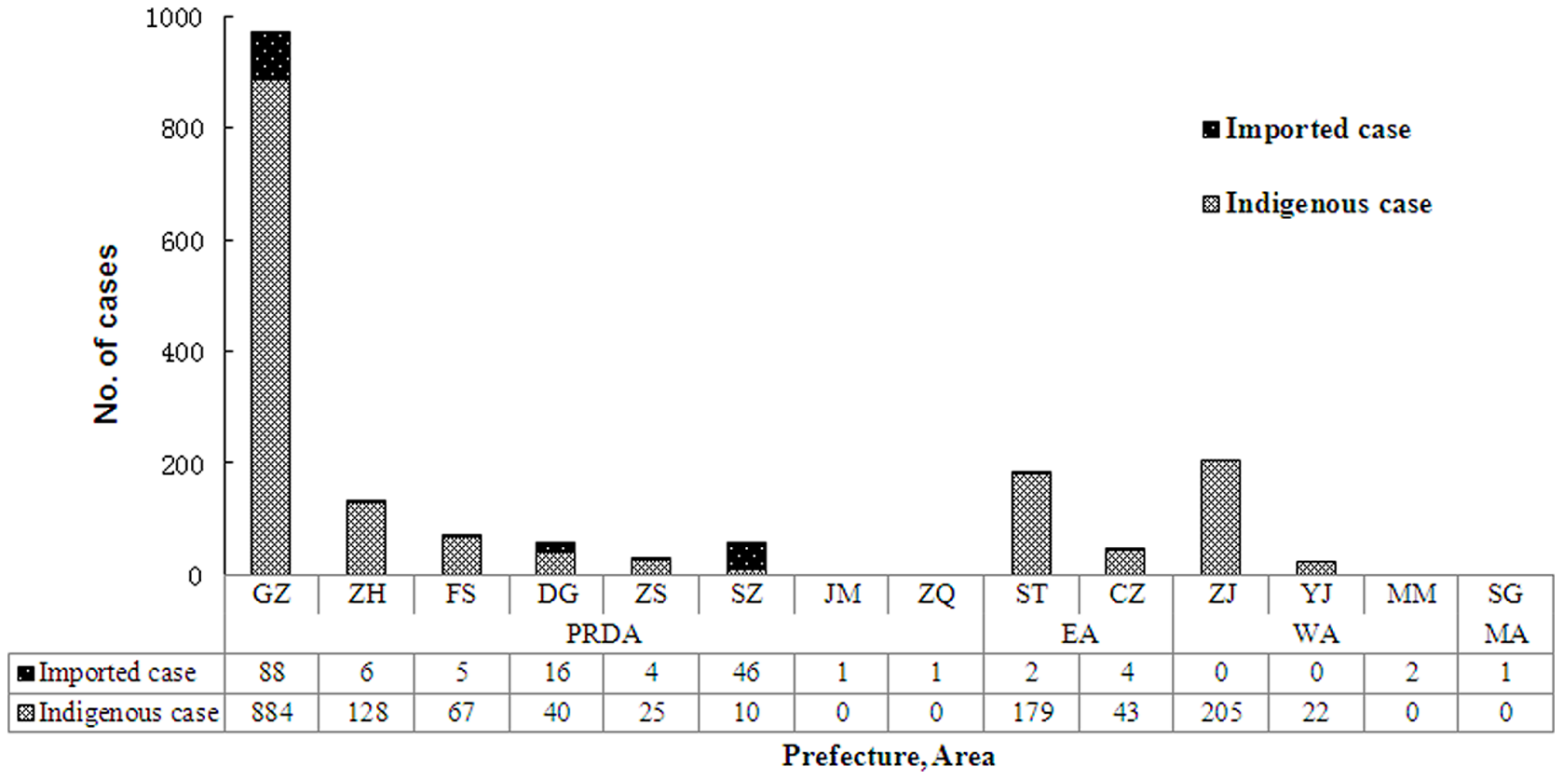
Spatial distribution of dengue cases by infection origin in Guangdong, China, 2005–2011. X-axis represents different prefectures (e.g. GZ,ZH,FS) and areas (PRDA, EA, WA, MA) of Guangdong province: Data tables were shown under the chart to display the case number of imported and indigenous cases. GZ (the metropolis capital city of Guangdong) in PRDA, ST in EA, and ZJ in WA were the top 3 clustered areas.

In total, 176 cases (9.89%) were imported and 1603 cases (90.11%) were indigenous. Imported cases were detected throughout the year with a sporadic distribution pattern, whereas indigenous cases were concentrated in July–November during which period 99.06% (1588/1603) of the indigenous cases occurred. A spatial distribution analysis of indigenous cases found that 71.9% (1153/1603) of these cases were identified in PDRA, with the metropolis capital city Guangzhou as a high-risk center (comprising 55.1%, 884/1603). Zhan-jiang prefecture in WA and Shan-tou prefecture in EA were the other 2 clustered areas, respectively accounting for 12.8% (205/1603) and 11.2% (179/1603; Figures 2 and 3).

### Clinical features: the vast majority of cases presented with symptoms of mild and typical dengue fever

**Figure 4:**
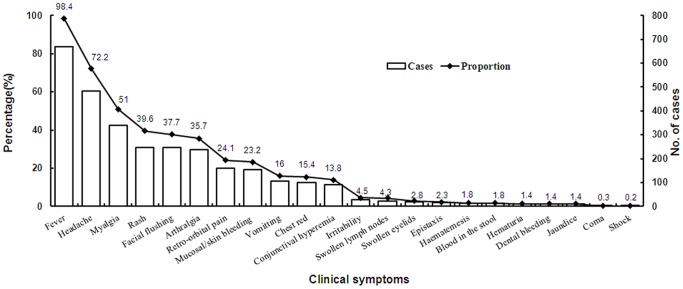
Clinical presentations of dengue infections in Guangdong, China, 2005–2011. Percentage of certain clinical symptom was shown with the line chart. Case number was shown with the bar chart. Data labels of percentage were appended to the line chart.

A total of 686 dengue patients who were administered the detailed questionnaires underwent clinical spectrum analysis. The majority of these patients presented with typical or mild manifestations, with a sudden fever (98.4%), headache (72.2%), myalgia (51.0%), and rash (39.6%) being the primary clinical symptoms. The mean axillary temperature was 38.9°C. In total, 81.4% (322/394) of the patients had leukopenia and 71.4% (275/385) had thrombocytopenia (Figure 4).

There were a small number of severe cases that presented with epistaxis (2.3%), hematemesis (1.8%), blood in the stool (1.8%), hematuria (1.4%), dental bleeding (1.4%), coma (0.3%), and shock (0.2%); however, no cases of dengue hemorrhagic fever, dengue shock syndrome [22], [23], or death were reported between 2005–2011.

### Seroprevalence of DENV specific IgG in healthy population

**Figure 5:**
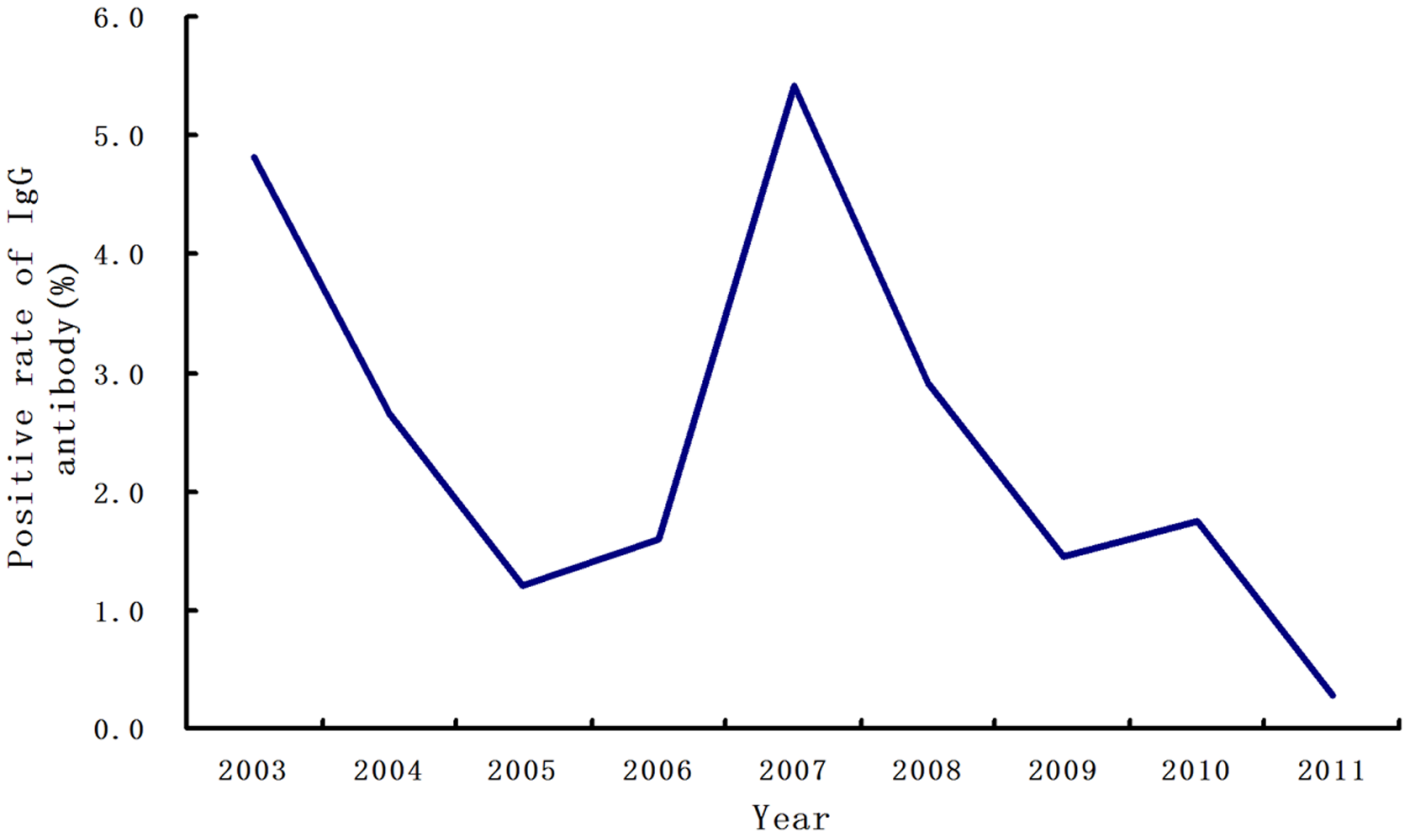
Seroprevalence of specific immunoglobulin (Ig) G antibody against dengue virus among healthy inhabitants. Data were collected through 2003–2011 in 4 sentinel cities of Guangdong, south China. Seroprevalence rate of IgG antibody by year was shown with the line chart. The average positive rate of IgG antibody was 2.43%.

A total of 5,586 sera samples from local healthy inhabitants were collected during 2003–2011 in Guangdong. The overall positive rate of IgG antibody was 2.43% (136/5586). The year 2007 had the highest rate of 5.42% (45/830), followed by 2003 with 4.81% (9/187), 2008 with 2.91% (29/995), and 2004 with 2.65% (18/680). For all other years, the IgG antibody positivity rate remained below 2%. Two small conspicuous peaks were observed, in 2003 and 2007, followed by a diminishing trend in the other years (Figure 5).

### Etiological surveillance results

**Figure 6:**
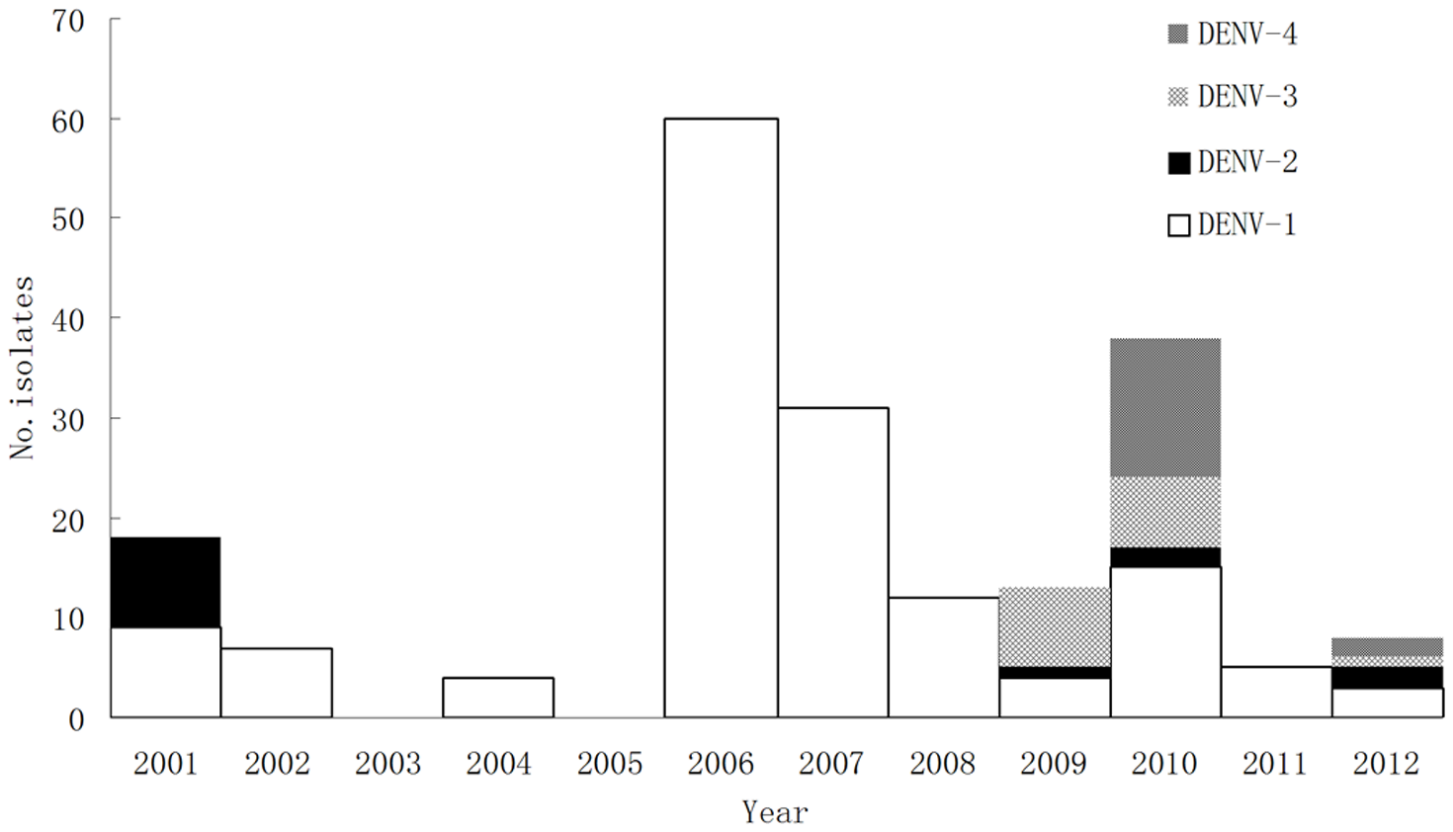
Serotypes of dengue virus identified in autonomous dengue patients from outbreak localities of Guangdong. Etiology data were collected from 2001 to 2012 (as of September 15, 2012). No viruses were isolated from indigenous cases in 2003 and 2005. Stacked bar chart was used to show the different serotypes of dengue viruses with different filling patterns.

A total of 1603 indigenous cases were detected from 2006 to 2011. Only imported cases were identified in 2005. Of the 337 acute-phase sera analyzed, 188 were positive for virus isolation or PCR nucleic acid detection. DENV-1 was the predominant serotype in 78.2% of positive specimens (147/188). Before 2009, DENV-1 was the only serotype detected in indigenous dengue cases, except in 2001 when DENV-1 and DENV-2 were both isolated in different prefectures of Guangdong. From 2009 to 2012, all 4 serotypes (DENV-1, DENV-2, DENV-3, and DENV-4) were derived from autonomous patients from different outbreak localities (as of September 15, 2012; Figure 6).

## Discussion

Our epidemiological, serological, and etiological study during 2005–2011 provided a good understanding of the current epidemiological situation of dengue infections in Guangdong. In spite of the exposure to a weak force of infection, as reflected in the low seroprevalence of the IgG antibody, the predominant circulation of DENV-1 for consecutive years and the co-existence of multiple serotypes since 2009 indicates the endemicity of dengue infections in Guangdong.

This study showed that there was no increased risk of acquiring dengue infections among younger individuals, and adults aged 20–59 years had a comparatively higher incidence of infection; this finding is different from the age distribution pattern observed in many dengue-endemic areas, where the shift towards a higher incidence in younger patients was reported [3], [25]. However, this may still be a cause for concern, as dengue can be endemic in areas where a great number of adults are affected. It is also noteworthy that the periodic outbreaks and epidemics in Guangdong in recent years were largely led by the predominant DENV-1 serotype, which is considered to have a much lower risk of causing severe dengue compared to DENV-2, DENV-3, and DENV-4 [26], [27]. This finding might explain why the vast majority of dengue infections in Guangdong presented as mild or typical dengue fever instead of severe forms of the disease.

It is notable that the years 2003 and 2007 had a relatively higher seroprevalence of IgG antibody against DENV than the other years included in this study, indicating an unusual circulation state of DENV during those years. This can be explained by the large-scale outbreaks and epidemics in preceding years (in 2002 and 2006, respectively) in PDRA, WA, and EA of Guangdong province [28], [29]. Fortunately, the overall low seroprevalence of IgG antibody (<5%) in healthy populations suggested the presence of an average weak force of infections in Guangdong.

The epidemic pattern of dengue in Guangdong to date is seemingly different from that in hyper-endemic areas, given the age profile, clinical spectrum of patients, and the low seroprevalence of the dengue IgG antibody. However, the monotypic circulation state for continuous years may presage the hypo-endemicity of dengue infections. In addition, more evidence in favor of endemicity has been observed since 2009. Multiple serotypes of viruses were identified in outbreak localities [15], [30]–[33], changing the monotypic situation led by the predominant DENV-1 serotype for 7 years (from 2002–2008). In 2010 and 2012, all 4 serotypes were detected one after another in indigenous outbreak locations.

These results remind us of the possibility of a changing circulation pattern in Guangdong, from non-endemicity (no virus sustained), to hypo-endemicity (one serotype present), and even hyper-endemicity (multiple serotype present) of dengue infections [21], [34]. It is important to have a clear understanding of the current dengue epidemic in Guangdong, which is the most densely populated province in southern China, surrounded by large number of dengue-endemic countries, and which has a subtropical climate, providing the optimal environmental, social, and biological circumstances for vector mosquito breeding and reproduction [1]. The change from monotypic to multitypic circulation of DENV in previous years might have an important bearing on the epidemiology of dengue in Guangdong, especially for the capital city of Guangzhou. As the hardest hit location in terms of reported cases and clustered areas, Guangzhou is confronted with a particular challenge for the prevention and control of dengue transmission, particularly taking into account the favorable factors of a dense population and a high degree of urbanization.

Through the analysis of the characteristics of dengue infections in Guangdong, our findings affirm the endemic nature of this disease on the basis of the patients, virus, and serology surveillance in recent years. However, we have to address several limitations of our study. Firstly, patients were identified through the National Notifiable Disease Reporting Network, which mainly relies on the reporting of diseases by medical practitioners. Although this passive surveillance network covers over 99.5% of the hospitals and clinics in the province, the possible omission of cases associated with healthcare providers (due to awareness of diagnosis and reporting and a sense of responsibility) could not be completely excluded. However, the impact on the reliability of our data owing to self-reporting should be very small. Secondly, we cannot obtain age-specific seroprevalence rates for dengue, owing to the lack of age stratification during sampling. A more stringently designed serology survey (with age-stratified sampling and a bigger sample size) is needed to have a better assessment of infection status. Thirdly, the majority of sera samples were collected beyond the acute phase of dengue infection, resulting in a lack of acute-phase samples for etiology diagnostic testing; an even smaller percentage of samples were obtained for further molecular evolution analysis. Fourthly, the lack of phylogenetic analysis for viral isolates from local places and surrounding dengue-endemic countries made it difficult to understand their phylogenetic relationships and to determine the source of infections.

Although it has its limitations, our study helps to illustrate the epidemiological situation of dengue infection in Guangdong, China. Our findings describing the endemic nature in the last several years will have important implications for the prevention and control of dengue in the province. A well-designed serological and etiological surveillance program targeting patients, the healthy population, and vector mosquitos should be considered. Furthermore, phylogeographic analysis of viral isolates derived from outbreak localities in Guangdong and the surrounding Southeast Asian countries is needed to clearly determine the evolutionary relationship of dengue viruses.
